# CD317 maintains proteostasis and cell survival in response to proteasome inhibitors by targeting calnexin for RACK1-mediated autophagic degradation

**DOI:** 10.1038/s41419-023-05858-1

**Published:** 2023-05-20

**Authors:** Jian Cheng, Guizhong Zhang, Tian Deng, Zhao Liu, Mengqi Zhang, Pengchao Zhang, Funmilayo O. Adeshakin, Xiangyun Niu, Dehong Yan, Xiaochun Wan, Guang Yu

**Affiliations:** 1 Department of Immunology, Jinzhou Medical University, Jinzhou, Liaoning China; 2 Center for Protein and Cell-based Drugs, Institute of Biomedicine and Biotechnology, Shenzhen Institute of Advanced Technology, Chinese Academy of Sciences, 518055 Shenzhen, People’s Republic of China; 3 Guangdong immune cell therapy engineering and technology research center (No. 2580 [2018]), Shenzhen, China; 4 University of Chinese Academy of Sciences, 100049 Beijing, PR China

**Keywords:** Cancer therapeutic resistance, Chaperone-mediated autophagy, Oncogenes, Protein aggregation

## Abstract

Unbalanced protein homeostasis (proteostasis) networks are frequently linked to tumorigenesis, making cancer cells more susceptible to treatments that target proteostasis regulators. Proteasome inhibition is the first licensed proteostasis-targeting therapeutic strategy, and has been proven effective in hematological malignancy patients. However, drug resistance almost inevitably develops, pressing for a better understanding of the mechanisms that preserve proteostasis in tumor cells. Here we report that CD317, a tumor-targeting antigen with a unique topology, was upregulated in hematological malignancies and preserved proteostasis and cell viability in response to proteasome inhibitors (PIs). Knocking down CD317 lowered Ca^2+^ levels in the endoplasmic reticulum (ER), promoting PIs-induced proteostasis failure and cell death. Mechanistically, CD317 interacted with calnexin (CNX), an ER chaperone protein that limits calcium refilling via the Ca^2+^ pump SERCA, thereby subjecting CNX to RACK1-mediated autophagic degradation. As a result, CD317 decreased the level of CNX protein, coordinating Ca^2+^ uptake and thus favoring protein folding and quality control in the ER lumen. Our findings reveal a previously unrecognized role of CD317 in proteostasis control and imply that CD317 could be a promising target for resolving PIs resistance in the clinic.

## Introduction

Proteins, accounting for more than 50% of the dry mass of most cells, are involved in practically every biological process [1]. Proteostasis (sometimes called proteome homeostasis) is thus a prerequisite for cellular and organismal health [1]. Due to inevasible unfavorable conditions (hypoxia, glucose deprivation, lactic acidosis, oxidative stress, insufficient amino acid supplies, etc.) and the intrinsic stresses (oncogene activation, changes in chromosomal number, and increased glycolysis, etc.), tumorigenesis is frequently associated with an unbalanced proteostasis network, which sensitizes cancer cells to drugs targeting proteostasis regulators [2–4].

The proteasome is a multi-subunit protease that destroys polyubiquitinated proteins, the majority of which are misfolded or unassembled polypeptides, making it a key regulator of proteostasis [5]. Tumor cells, owing to their altered proteome landscape, appear to rely more on the proteostasis network for survival and proliferation than normal cells, a phenomenon known as proteostasis addiction [5, 6]. As a result, proteasome inhibitors (PIs), when correctly dosed, can kill cancer cells while sparing normal cells, and hence have been approved by FDA for curing multiple myeloma and mantle cell lymphoma [7]. Despite their early success, PIs have modest activity against solid tumors [5], and they frequently fail to cure hematological malignancies due to acquired resistance [8]. Drugs that target other components or regulators of proteostasis are thus required to overcome such limitations of PIs.

CD317, also known as BST2, tetherin, or HM l.24, is a type II transmembrane glycoprotein that localizes to the cell surface and several intracellular compartments [9, 10]. It has been linked to a variety of physiological and pathological processes, including viral particle tethering, inflammation, and immunological modulation, as well as tumorigenesis [11–13]. CD317 is overexpressed in a variety of malignancies [12] and activates multiple signaling axes (EGFR, ERK, NF-κB, RICH2/Cytoskeleton, etc.) to promote proliferation, migration, drug- and immune cytolysis-resistance, and other cellular processes [14–18]. In animal models, neutralizing monoclonal antibodies, shRNAs or peptides targeting CD317 reduced tumor growth [17, 19–21]. Furthermore, CD317 is implicated in IRE1α-mediated ER stress in influenza A virus-infected host cells, according to a recent study [22], indicating that CD317 could be a potential proteostasis regulator. However, it is unknown whether CD317 affords an advantage to proteostasis regulation in tumor cells and if so, what the underlying molecular mechanism is.

In this study, we investigate the role of CD317 in tumor proteostasis. We found that CD317 is upregulated in several hematological malignancies and affords a survival advantage to tumor cells in response to PIs. Interestingly, the impact of CD317 is mediated by calnexin (CNX), an ER chaperone protein that negatively regulates ER Ca^2+^ capacity by inhibiting calcium refilling through Ca^2+^ pump SERCA [23, 24]. CD317 interacts with CNX and subjects it to RACK1-mediated autophagic degradation, orchestrating Ca^2+^ uptake and protein folding in the ER lumen. These findings suggest that CD317 plays an important role in proteostasis control and could be used to improve the therapeutic efficacy of PIs as well as other proteostasis-targeting drugs.

## Materials and methods

### Reagents

DMEM, RPMI-1640 medium, and fetal bovine serum (FBS) were purchased from HyClone, L-glutamine from Gibco, Propidium Iodide (PI) Solution (421301) from Biolegend (CA, USA), Annexin V-FITC/PI apoptosis detection kit (FA101-02) from TransGen Biotech (Beijing, China), Lipofectamine 3000 (L3000015) from Invitrogen (CA, USA), Thapsigargin (CHXSC0389), MG-132 (S1748), Fura-2 AM (S1052) and Fluo-4 AM (S1060) from Beyotime (Jiangsu, China), Chloroquine (CQ, 105M4035V) from Sigma, Bortezomib (PS-341, S1013), BafilomycinA1 (BAF, S1413) from Selleckchem (PA, USA), Cycloheximide (CHX, C112766) from Aladdin (Shanghai, China).

### Bioinformatics analysis

CD317 expression in hematological cancers was determined by analysis of Piccaluga lymphoma, Maia/Choi/Stegmaier Leukemia (20979_at), and Zhan Myeloma (Y12856_at) datasets, which are available through Oncomine (www.oncomine.org). CD317 expression in the cell lines derived from hematologic malignancies was determined through analysis of RNA-seq data from the Cancer Cell Line Encyclopedia (https://sites.broadinstitute.org/ccle/).

The association between CD317 or CNX expression in Acute Myeloid Leukemia (AML, LAML) and survival prognosis was analyzed using the Oncolnc database (http://www.oncolnc.org/). Lower and upper percentiles were 60% and 40%, respectively.

### Cell culture

HeLa, K562, Jurkat, and NCI-H929 cells with STR profiling were purchased from the Shanghai Cell Bank of the Chinese Academy of Sciences (Shanghai, China) or ATCC and stored in-house. Cell lines were cultured in DMEM or RPMI-1640 medium (HyClone) supplemented with 10% FBS (HyClone) and 2 mmol/L l-glutamine (Gibco), and checked for the absence of *Mycoplasma* by PCR.

### Transfection

Transfection of cells with plasmids or siRNAs was performed using Lipofectamine 3000 (Invitrogen, Carlsbad, CA, USA) or RFect Transfection Reagent (Cat#:11026, 21015, Baidai biotechnology, Changzhou, China) according to the manufacturer’s protocol. Human CD317-specific siRNAs and siRNA-resistant (SR) CD317, delCT, and delGPI constructs (they are called HA-CD317-SR, HA-delCT-SR, and HA-delGPI-SR, respectively) were described previously [17]. Calnexin-Myc-His (Myc-CNX) and RACK1-Flag-His (Flag-RACK1) plasmids were purchased from WZ Bioscience Inc. Human Calnexin siRNAs, and RACK1 siRNAs were described in refs. [25] and [26], respectively. Table 1 lists their corresponding sequences.

**Table 1:** siRNA sequences.

siRNA	Sequence (5’ → 3’)
siCNX-1	AAGACGATACCGATGATGAAA
siCNX-2	AATGTGGTGGTGCCTATGTGA
siRACK1-1	CCAUCAAGCUAUGGAAUACTT
siRACK1-2	GCUAUGGAAUACCCUGGGUTT

### Intracellular calcium assay

Fluorescence staining with either Fluo-4 AM or Fura-2 AM was used to measure intracellular calcium.

Forty-eight hours after transfection, cells were collected, washed three times with calcium-free PBS, and loaded with 1 μM Fluo-4 AM for 20 min at 37 °C. Then the cells were rinsed three times with PBS after removing the excess dye, and then incubated for another 20 min to ensure that Fluo-4 AM was entirely changed into Fluo-4. Cytosolic calcium ([Ca^2+^]_C_) was measured at room temperature using a flow cytometer (CytoFLEX, BECKMAN COULTER). Total ER calcium storages ([Ca^2+^]_ER_) were determined by measurement of ER calcium loss in response to Thapsigargin (TG, SC0389, Byotime, 1–2 μM), which inhibits SERCA2b-mediated calcium reuptake. The ratio of TG-induced fluorescent signals relative to baseline reflected [Ca^2+^]_ER_.

For Fura-2 AM fluorescence staining, the same number of harvested cells from each group were incubated with 4 μM Fura-2 AM (Beyotime) at 37 °C for 1 h. After being washed by PBS three times, cells were exited at both 340 nm and 380 nm, and their fluorescence emission intensity at 510 nm was determined using SpectraMax i3x Muti-Mode Microplate Detection System (Molecular Devices). Calcium levels are expressed as relative ratios of fluorescence emission at 340 nm/380 nm.

### Cell death

Thirty-six hours after transfection (if applicable), cells were seeded in triplicates in 12-well plates 2–4 × 10^5^ cells/well, and allowed to grow for another 24 h in the presence or absence of BTZ. Cells were harvested, washed with PBS, and stained with propidium iodide (PI) at room temperature for 15 min in the dark, or with Annexin V/PI (FA101-02, TransGen Biotech) according to the instructions of the manufacturer. Cell death was assessed by flow cytometry (CytoFLEX, BECKMAN).

### RNA isolation and qRT-PCR

Total RNA was extracted using TRIzol reagent (Invitrogen) and used to generate cDNA. Specific primers used for quantitative real-time PCR assays were synthesized by GENEWIZ, Inc. (Suzhou, China). Table 2 lists their corresponding sequences.

**Table 2:** Primers of qRT-PCR.

Primer	Sequence (5’ → 3’)
Bip-F	ACGGCAGCTGCTATTGCTTA
Bip-R	TCCATGACACGCTGGTCAAA
GRP94-F	TTTGTTGTCGTGGCTGTCTTC
GRP94-R	TGGCTTGATGCTGTGGTCTTT
HRD1-F	ACCAGCATCCCTAGCTCAGA
HRD1-R	GAGCTGGAGGCCTTTCCAT
CD317-F	CACACTGTGATGGCCCTAATG
CD317-R	GTCCGCGATTCTCACGCTT
Actin-F	ATTGGCAATGAGCGGTTCCG
Actin-R	AGGGCAGTGATCTCCTTCTG

### Protein extraction and immunoblotting

Total cell lysates were prepared by suspending cells in the RIPA buffer (Beyotime, China) supplemented with 1× complete protease inhibitors mixture and 1× phosphatase inhibitor (Roche). Protein concentration was determined by BCA assay (Pierce, Rockford, USA). Equal quantities of proteins were separated by SDS-PAGE, transferred to a PVDF membrane, and blotted with specific antibodies. Protein in the membrane was visualized by an enhanced chemiluminescence detection kit (Millipore, USA). Antibodies against the following proteins were used, with catalog numbers and sources listed: CD317 (1C12) [18] (in-house); GAPDH (MB001) (Bioworld Technology); β-actin (A5441) (Sigma); K48-Linkage-Specific Ubiquitin (ab140601) (Abcam); Calnexin (00050122) (Proteintech); CHOP (L63F7) (CST); β-tubulin (200608) (ZEN BIO); HRP-conjugated mouse anti-HA1.1 tag (901519) (Biolegend); HRP-conjugated mouse anti-Flag (200-303-383) from Rockland; HRP-conjugated goat anti-mouse IgG (074-1806) from KPL, and HRP-conjugated goat anti-rabbit IgG (E030120-02) from EARTHOX. Full and uncropped western blots are presented in Supplemental File (Supplementary Fig. S7).

### Co-immunoprecipitation (Co-IP)

HeLa or Jurkat cells were lysed in immunoprecipitation (IP) buffer (150 mM NaCl, 50 mM Tris-HCl, pH 7.4, 50 mM EDTA, 1.0% Nonidet P-40, 1 mM PMSF) supplemented with 1× complete protease inhibitors mixture (Roche). Immunoprecipitation was performed using protein-A/G Dynabeads (Pierce, 88803) coupled with 1 μg anti-CD317 (Proteintech, 13560-1-AP), anti-HA (Sigma, H3663), or anti-Flag (Sigma, F3165) specific antibodies, respectively. Normal mouse IgG (Santa Cruz, sc-2025) or normal rabbit IgG (Santa Cruz, sc-2027) was used as a negative control. The eluted proteins were fractionated by SDS/PAGE and detected by Immunoblotting as described.

### RNA sequencing

Total RNA extracted from K562 cells transfected with CD317-specific or control siRNAs was used for mRNA library preparation. Completed libraries were sequenced on an Illumina HiSeq instrument by GENEWIZ, Inc. (Suzhou, China). To mine the altered pathways, Gene set enrichment analysis (GSEA) was performed on RNA sequencing data by using the KEGG gene sets from the Molecular Signature Database (https://www.gsea-msigdb.org/gsea/msigdb/). The normalized enrichment score (NES) was generated and used to assess the effectiveness of each enrichment in identifying candidate gene sets that influence cellular processes.

### Cycloheximide chase assay

K562 or Jurkat cells were treated with 50 µg/mL cycloheximide (CHX) and then incubated at 37 °C for the periods indicated. Cell pellets were collected for protein degradation analysis using immunoblotting.

### LC-MS/MS search of CD317 binding candidates

Lysates of HEK293T cells expressing HA-CD317 were subjected to immunoprecipitation (IP) using an anti-HA antibody. IP samples were run with LC-MS/MS analysis by Luming Bio (Shanghai, China).

### Immunofluorescence and microscopy

In total, 1 × 10^4^ HeLa cells were cultured on cell climbing slices. Following incubation for 6 h with 100 nM BAF, cells were fixed in ice-cold 4% paraformaldehyde and permeabilized in PBS with 0.05% Triton X-100 and 3% BSA. Immunostaining was performed using rabbit anti-CNX antibody (Proteintech, 10427-2-ap) and mouse anti-CD317 antibody (in-house, ref. [26]) followed by incubation with anti-mouse IgG Alexa Fluor 488 (Beyotime, P0188), anti-rabbit IgG Cy3 (Beyotime, P0183). For monitoring the colocalization of CNX and autophagosomes, cells were transfected with RFP-LC3, fixed in ice-cold 4% paraformaldehyde, and permeabilized with 0.1% saponin (Beyotime, P0095). Then immunostaining for CNX was performed as aforementioned description. Finally, microscopy was performed using a STELLARIS 5 confocal microscope from Leica Microsystem (Mannheim, Germany).

### Human samples and immunohistochemistry

The tissue microarrays (HLymT060PT01) containing 52 T-cell lymphoma (TCL) specimens and 8 normal lymphoid samples were obtained from OUTDO BIOTECH (Shanghai, China) and used to study the expression and relationship of CD317 and CNX using Immunohistochemistry (IHC), which was approved by the Ethics Committee of Shenzhen Institutes of Advanced Technology (SIAT), Chinese Academy of Sciences.

IHC staining was performed as previously described [17] using rabbit anti-CD317 mAbs (Proteintech, 1356-1-AP) or rabbit anti-CNX mAbs (Proteintech, 10427-2-AP). The levels of staining were scored according to the intensity (no staining = 0, weak staining = 1, moderate staining = 2, strong staining = 3) and the number of stained cells (0% = 0, 1–25% = 1, 26–50% = 2, 51–75% = 3, 76–100% = 4). Final immunoreactive scores were calculated by multiplying the staining intensity by the number of stained cells, with 0 and 12 as the minimum and maximum values, respectively. The Mann–Whitney *U* test was used to evaluate the statistical significance of the results.

### Statistical analysis

All cell experiments were performed triplicate. Analyses were performed using GraphPad Prism Software (San Diego, CA). Data were expressed as mean ± SEM. The student’s *t* test or Mann–Whitney *U* test was used to compare continuous data for two groups. Pearson correlation co-efficiency was used to analyze the relationship between CNX expression and the CD317 staining levels in tissue sections. The long-rank test was used to examine the differences in patient survival across groups. *P* values of less than 0.05 were considered significant.

## Results

### CD317 is upregulated in hematological malignancies and inhibits PIs-induced cell death

**Fig. 1:**
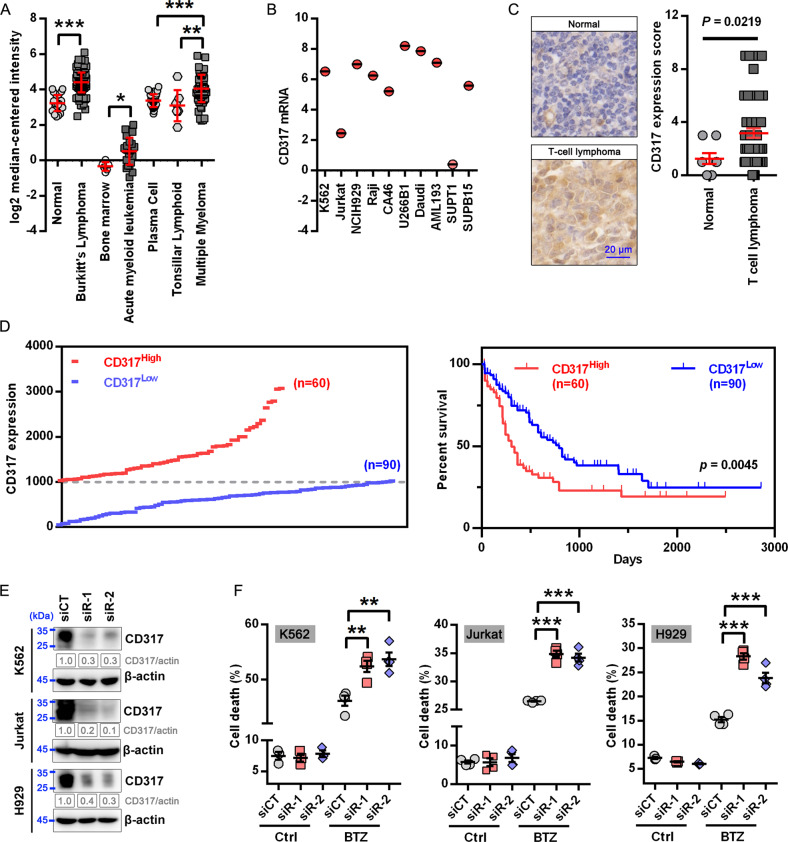
CD317 upregulation correlates with cell survival and poor prognosis in hematologic malignancies. **A** Oncomine data demonstrating upregulation of CD317 mRNA in multiple hematologic malignancies (HM). **B** CD317 mRNA levels in the human HM cell lines. Data were obtained from the CCLE database. **C** IHC analysis of CD317 protein expression in T-cell lymphoma (*n* = 52) and normal lymphoid samples (*n* = 8) by IHC. The quantitation of the CD317 expression was performed as described in Materials and Methods. Each data point represents a patient’s CD317 expression score. Scale bar, 20 μm. **D** The overall survival was compared between CD317 high (*n* = 60) and low expression (*n* = 90) in LAML patients from Oncolnc. **E** Immunoblot analysis of HM cells with or without CD317 knockdown. **F** FACS-based analysis of cell death in K562, Jurkat, and H929 cells that transiently transfected with CD317-specific siRNAs (siR-1 or siR-2) or control siRNAs (siCT) in the presence or absence of BTZ. *P < 0.05; **P < 0.01; ***P < 0.001.

To investigate the involvement of CD317 in hematological malignancies (HMs), we examined its expression by surveying the public database (Oncomine and CCLE). As seen in Fig. 1A, many HM samples, including those from Burkitt’s lymphoma [BL], acute myeloid leukemia [AML], and multiple myeloma [MM], had much higher levels of CD317 than their corresponding normal samples. Several HM cell lines were also found to harbor high levels of CD317 (Fig. 1B). In addition, we examined the expression of CD317 by IHC in 52 T-cell lymphomas (TCL) samples and 8 normal lymphoid samples, and found that CD317 protein was markedly elevated in the TCL samples (Fig. 1C). Using the Oncolnc database for survival analysis, we also discovered that AML patients with high CD317 expression (CD317^High^) have shorter overall survival times than those with low CD317 expression (CD317^Low^) (Fig. 1D), indicating that elevated levels of CD317 contribute to tumor progression, and serve as a key indicator for poor prognosis in HMs.

To test the function of CD317 in HMs cells, we used three cell lines: K562, Jurkat, and H929, which express low to high levels of endogenous CD317 mRNA (Fig. 1B). We knocked down CD317 in these cells using siRNAs (Fig. 1E). Although CD317 knockdown had no effect on cell proliferation (Supplementary Fig. S1A) or apoptosis in normal conditions (Fig. 1F and Supplementary Fig. S1B–D), it significantly increased cell death in response to the FDA-approved proteasome inhibitor Bortezomib (BTZ), suggesting that cells with low levels of CD317 have a fragile proteostasis. Moreover, the level of cell death was nearly restored to that in control cells when an siRNA-resistant form of CD317 was expressed in the CD317-knockdown cells (Supplementary Fig. S1E–F), confirming the specificity of the CD317 siRNAs. These findings suggest that CD317 is upregulated in HMs and is required for cell survival when proteostasis is challenged.

Not only in HM cells but also in several solid tumor cells, we discovered that knocking down CD317 increases BTZ-induced cell death (Supplementary Fig. S2A–D), and this effect can be reversed by re-expressing an siRNA-resistant form of CD317 (Supplementary Fig. S2E), demonstrating that CD317 is necessary for solid tumor cells to maintain proteostasis as well.

### CD317 silencing promotes PIs-induced proteostasis collapse

**Fig. 2:**
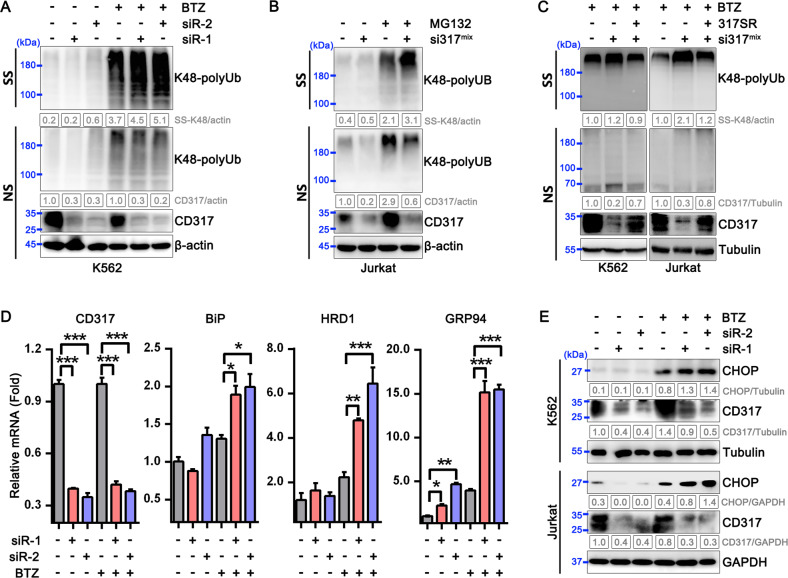
CD317 knockdown promotes PI-induced proteostasis collapse. **A** Immunoblot analysis of K48 polyUb-modified proteins in control and CD317-knockdown K562 cells treated with or without BTZ. NS, NP-40 soluble; SS, NP-40 insoluble but SDS soluble. **B** K48 polyUb-modified protein levels in control and CD317-knockdown Jurkat cells treated with MG-132. **C** K48 polyUb-modified proteins in CD317-knockdown K562 cells and Jurkat cells after forced expression of siRNA-resistant CD317. **D** Real-time PCR analysis of CD317, BiP, HRD1, and GRP94 mRNA levels in K562 cells. K562 cells were transfected with either CD317 siRNAs (siR-1 or siR-2) or control siRNAs. Forty-eight hours later, cells were treated with 1 μM BTZ for another 4 h before being collected for RNA extraction and subsequent real-time PCR assay. *P < 0.05; **P < 0.01; ***P < 0.001. **E** Immunoblot analysis of CHOP expression in K562 and Jurkat cells. 48 hours after transfection (CD317 siRNAs or control siRNAs), cells were treated with 1 μM BTZ for an additional 6–8 h before being harvested for immunoblotting.

Since CD317 protects cells from PIs-induced cell death, we looked at how it affects proteostasis. In response to PIs, CD317-knockdown cells display a fragile global proteostasis, as evidenced by a noticeable increase in the quantities of ubiquitinated misfolded proteins, as compared to their corresponding control cells (Fig. 2A–C and Supplementary Fig. S2F). Forced expression of the siRNA-resistant CD317, on the other hand, prevented the collapse of proteostasis in CD317-knockdown cells (Fig. 2C and Supplementary Fig. S2G). Furthermore, CD317 knockdown increased expression of the two conventional adaptive ER stress markers Bip and GRP94, the polytopic dislocon HRD1, as well as the maladaptive ER stress marker CHOP (Fig. 2D, E), which is linked to apoptosis. These data indicate that a reduction of CD317 increased misfolded proteins, ER stress, and even cell death.

### CD317 silencing disrupts intracellular calcium homeostasis

**Fig. 3:**
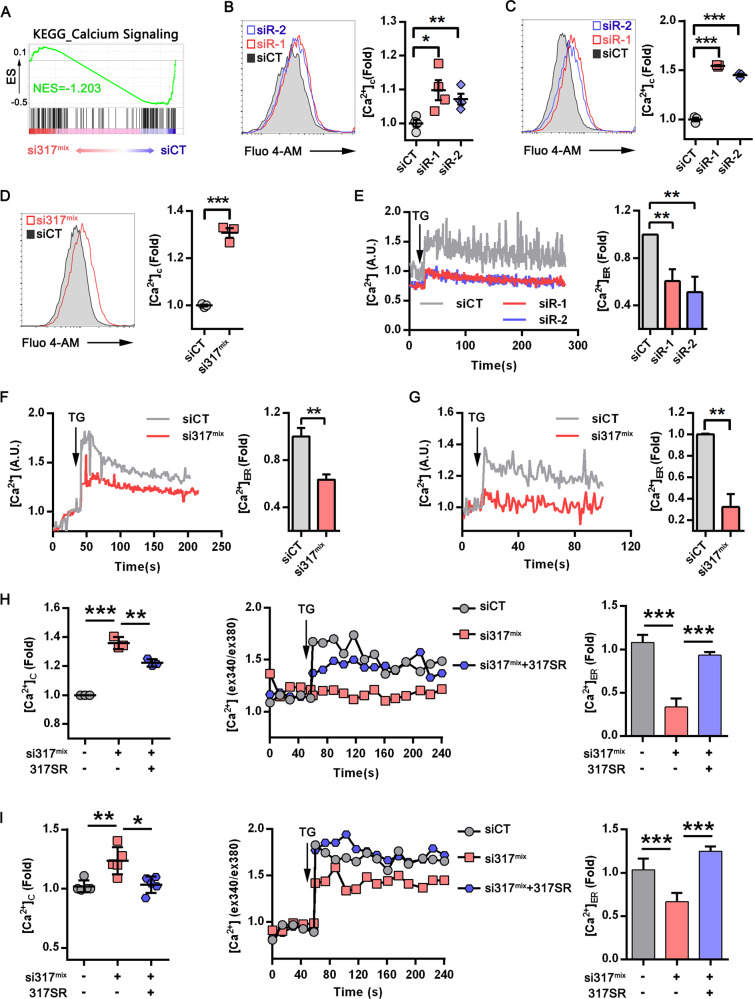
CD317 is implicated in calcium homeostasis maintenance. **A** GSEA enrichment plot of KEGG calcium signaling genes in control (siCT) versus CD317-knockdown K562 cells (si317^mix^); **B**–**D** Representative FACS graphs (left) and quantification (right) of cytosolic calcium ([Ca^2+^]_C_) in CD317-knockdown K562 (**B**), Jurkat (**C**), H929 (**D**), and their corresponding control cells. *P < 0.05; **P < 0.01; ***P < 0.001. **E**–**G** Representative FACS graphs (left) and quantification (right) of ER calcium ([Ca^2+^]_ER_) in CD317-knockdown K562 (**B**), Jurkat (**C**), H929 (**D**), and their corresponding control cells; TG, Thapsigargin; **P < 0.01. **H**, **I** Fluorescence measurements of intracellular calcium in CD317-knockdown K562 cells (**H**) and Jurkat cells (**I**) after forced expression of siRNA-resistant CD317 using Fura-2 AM staining. *P < 0.05; **P < 0.01; ***P < 0.001.

The mechanism by which CD317 supports proteostasis was then studied. We discovered that knocking down CD317 substantially reduces the calcium signaling utilizing gene set enrichment analysis of RNA-seq transcriptomics (Fig. 3A). CD317 knockdown, in particular, caused a noticeable rise in cytosolic Ca^2+^ (Fig. 3B–D) and a marked reduction of ER Ca^2+^ (Fig. 3E–G), demonstrating that CD317 downregulation disrupts calcium homeostasis. This calcium imbalance (lower ER calcium levels and aberrant cytosol calcium levels) has the potential to impair ER proteostasis by altering the action of diverse ER chaperones involved in protein folding and quality control machineries, ultimately leading to ER stress and apoptosis. Forced expression of an siRNA-resistant form of CD317 in CD317-knockdown cells significantly alleviated calcium and protein disorders (Figs. 3H, I and 2C), demonstrating that CD317 is necessary for preserving proteostasis and calcium homeostasis.

### CD317 interacts with CNX and is essential for its stability

**Fig. 4:**
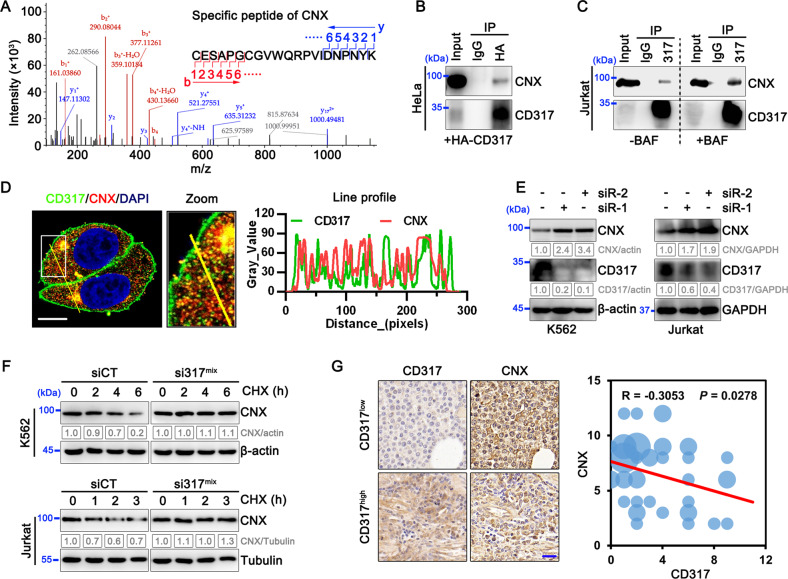
CD317 interacts with CNX and is essential for its stability. **A** LC-MS/MS fragment spectrum of the CNX-specific peptide. **B** Co-immunoprecipitation (IP) of endogenous CNX proteins with HA-CD317. Lysates of HA-CD317-transfected HeLa cells were immunoprecipitated with anti-HA antibodies as indicated. Cell lysates (input) and immunoprecipitates were analyzed by immunoblotting using CD317 mAbs and CNX mAbs. **C** Co-immunoprecipitation of CNX proteins with endogenous CD317. Prior to IP of CD317, Jurkat cells were treated for 6 h with or without 100 nM BAF, and the interaction of CNX with CD317 was determined using immunoblotting. **D** Immunofluorescence of CD317 and CNX in HeLa cells after 6 h of treatment with 100 nM BAF. Nuclei are stained blue with DAPI. Colocalization was analyzed using the RGB profiler in ImageJ and was indicated by the similarity of the patterns of red and green peaks along with the line distance; Scale bars: 10 μm. **E** Immunoblot analysis of CNX and CD317 proteins in the indicated cells transfected with CD317 siRNAs (siR-1 or siR-2). **F** Degradation of CNX in CD317-knockdown K562 (upper), Jurkat (bottom) and their corresponding control cells determined by Cycloheximide (CHX) chase assays. **G** Immunohistological staining (left) and correlation co-efficiency (right) of CD317 and CNX in T-cell lymphoma specimens. The scale bar represents 20 μm.

To figure out how CD317 influences calcium homeostasis, we conducted immunoprecipitation (IP) coupled with LC-MS/MS to discover targets that bind to CD317. Calnexin (CNX), a lectin chaperone that not only aids protein folding and quality control but also interacts with SERCA to limit ER calcium waves [23], was identified as a possible interacting candidate (Fig. 4A). Through Co-IP experiments in HeLa and Jurkat cells, the interactions of exogenous and endogenous CD317 with CNX were further confirmed (Fig. 4B, C). In addition, we mapped the CNX-binding site in CD317 and discovered that the CD317-CNX interaction depends on the C-terminal glycophosphatidylinositol (GPI) anchor rather than the N-terminal cytoplasmic tail (CT) domain (Supplementary Fig. S3A, B). In light of this, molecular modeling also revealed the potential for the C-terminal GPI domain of CD317 to interacted with CNX (Supplementary Fig. S3C). CD317 is a lipid raft-anchored protein, whereas CNX is an ER protein. To understand how they interact together to drive calcium homeostasis, we employed confocal microscopy to examine the intracellular distribution of CD317 and CNX. Interestingly, endogenous CD317 was identified not only on the cell membrane but also throughout the intracellular compartments, where it co-localized with endogenous CNX (Fig. 4D), which raises the intriguing possibility that CD317 may target CNX intracellularly.

To study the physiological role of CD317-CNX interaction, we knocked down CD317 expression in K562 and Jurkat cells and found that doing so significantly increased CNX protein levels by retarding CNX degradation (Fig. 4E, F). To corroborate the regulation of CD317 in CNX protein degradation in human malignancies, we analyzed the levels of CD317 and CNX in 52 TCL samples and found CNX protein levels were inversely correlated with CD317 expression (Fig. 4G). According to a survival analysis from Oncolnc database, better clinical outcomes are linked to AML patients’ higher CNX expression (Supplementary Fig. S4). This implies that CNX suppression caused by CD317 may promote tumor progression.

### CD317 targets CNX to RACK1-mediated autophagic degradation

**Fig. 5:**
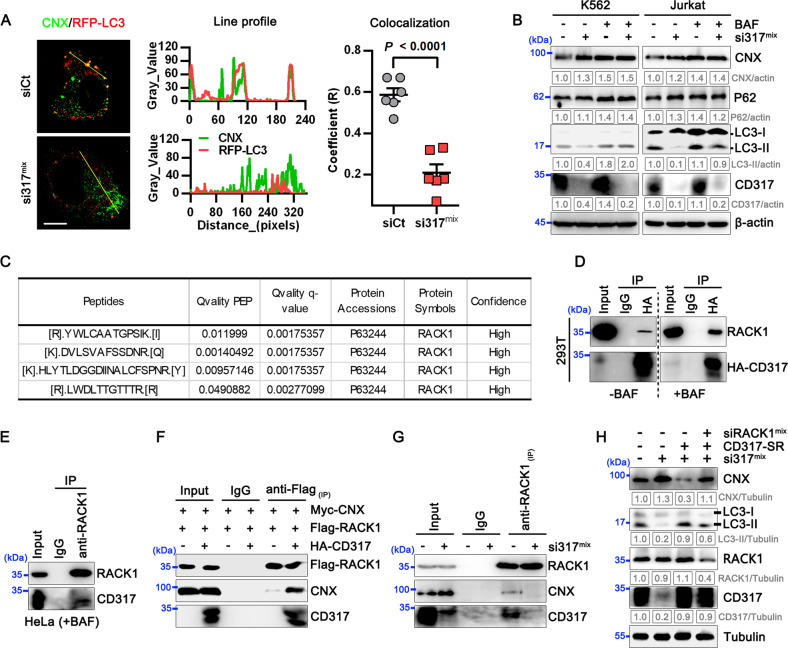
CD317 drives CNX degradation via RACK1-mediated autophagy. **A** Immunofluorescence of CNX and RFP-LC3 in CD317-knockdown HeLa (si317^mix^), and the control cells (siCT). Local and global colocalizations were determined by the line intensity fluorescent profile and Pearson’s correlation coefficient (R), respectively. Scale bars: 10 μm. **B** Immunoblot analysis of CNX proteins in cells treated with or without BAF as indicated. **C** Peptide bioanalysis using LC-MS/MS identifies RACK1 as a CD317-interacted candidate. **D** Co-IP analysis of exogenous RACK1 proteins with HA-CD317. Lysates of HEK293T cells transfected with HA-CD317 plus Flag-RACK1 plasmids were immunoprecipitated using anti-HA antibodies. Cell lysates (input) and immunoprecipitates were assessed by immunoblotting using CD317 mAbs and RACK1 mAbs. **E** Immunoblot analysis of the interaction between endogenous RACK1 and CD317 in HeLa cells. **F** Immunoblot analysis of the interaction between CNX, CD317, and RACK1 in HEK293T cell lysates after IP with anti-Flag mAbs. **G** Immunoblot analysis of the endogenous interaction between CNX, CD317, and RACK1 in HeLa cells transfected with either CD317 siRNAs or control siRNAs. **H** Immunoblot analysis of the indicated proteins in CD317-knockdown HeLa cells transfected with siRNA-resistant (SR) CD317 plasmids together with RACK1 siRNAs or control siRNAs.

CD317 has been implicated in protein degradation by direct interaction and subsequent selective autophagy [27, 28]. CD317 binds to CNX, and their interaction noticeably increased in response to the autophagy inhibitor BAF (Fig. 4C), suggesting that CD317 may target CNX to selective autophagic degradation. Therefore, we first confirmed whether CD317 is involved in autophagy and found that CD317-knockdown cells showed lower levels of autophagy (Supplementary Fig. S5A–C). Although CNX is not subject to autophagic degradation in CD317-negative HEK293 cells [29], we observed a noticeable colocalization of CNX with autophagic vesicles in CD317-positive HeLa cells, which is markedly reduced when CD317 is knocked down (Fig. 5A). Moreover, autophagy blockade with BAF treatment led to a dramatic rise in CNX, essentially eliminating the CNX discrepancy between CD317-knockdown and their comparable cells (Fig. 5B). In contrast, autophagy activation by Rapamycin resulted in a significant reduction in CNX protein levels, and CD317 was no longer able to affect CNX expression in this circumstance (Supplementary Fig. S5D). These findings suggest that CD317 drives CNX to autophagic degradation.

RACK1, a recently characterized dynamic component of the core autophagy machinery [30, 31], is another potential CD317 interacting candidate according to our previous interactome test (Fig. 5C). Immunoprecipitation assays supported the findings. CD317 did interact with RACK1, and their interaction was prominently increased after cells were treated with autophagy inhibitors BAF (Fig. 5D, E), implying that the CD317-RACK1 interaction is implicated in autophagy. Furthermore, CD317-RACK1 interaction is dependent on the C-terminal GPI anchor rather than the N-terminal CT domain (Supplementary Fig. S3B, C). We also detected the interaction between RACK1 and CNX and found essentially minimal interaction in the absence of CD317. In contrast, CD317 dramatically improved the RACK1-CNX interaction (Fig. 5F). Moreover, CD317 knockdown markedly impaired the endogenous interaction between RACK1 and CNX in HeLa cells (Fig. 5G). To explore whether RACK1 is required for CD317 function in CNX degradation, we detected CNX expression by western blot analysis and found that forced expression of an siRNA-resistant form of CD317 in CD317-knockdown cells effectively restored CNX expression to that of control cells (Fig. 5H). RACK1 knockdown, on the other hand, impaired such function of CD317 (Fig. 5H), confirming that RACK1 is indispensable for CD317-mediated CNX autophagic degradation.

Taken together, these results indicate that CD317 acts as a scaffold connecting CNX to RACK1 and subsequent autophagy.

### CNX silencing mitigates CD317 downregulation-induced calcium disorder, proteostasis collapse, and subsequent cell death

**Fig. 6:**
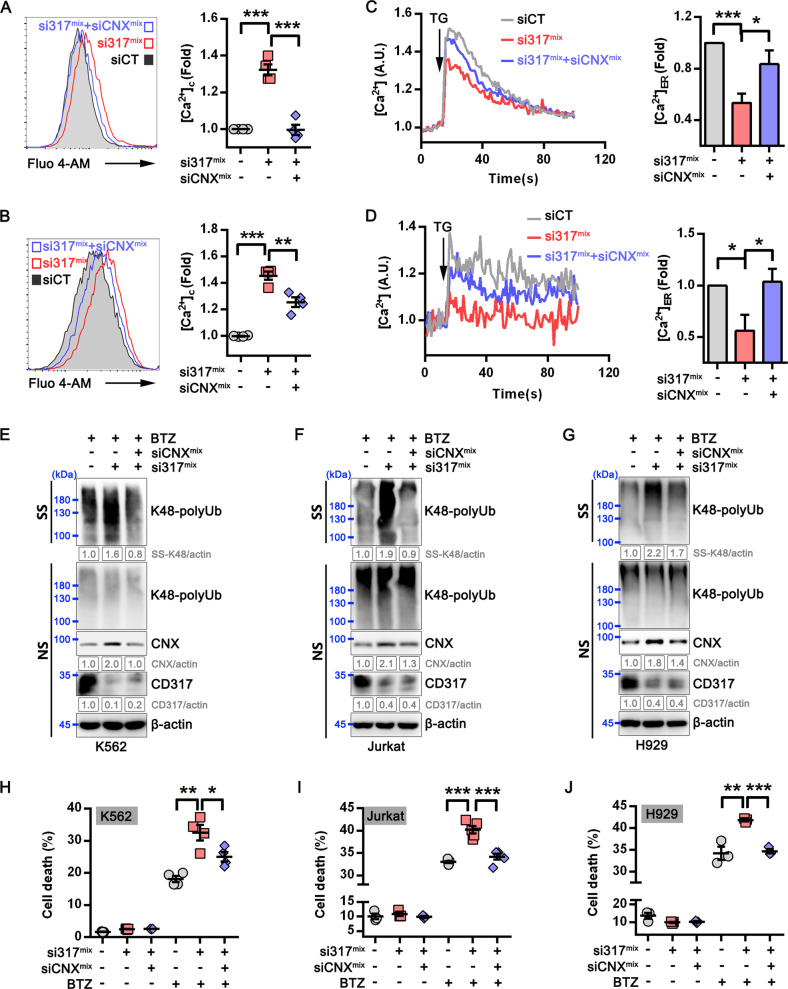
CNX is indispensable for CD317-mediated cell homeostasis and survival. **A**, **B** Representative FACS graphs (left) and quantification (right) of cytosolic calcium ([Ca^2+^]_C_) in K562 (**A**) and Jurkat (**B**) cells transfected with indicated siRNAs. **P < 0.01; ***P < 0.001. **C**, **D** Representative FACS graphs (left) and quantification (right) of ER calcium ([Ca^2+^]_ER_) in K562 (**C**) and Jurkat (**D**) treated with indicated siRNAs; TG, Thapsigargin; *P < 0.05, ***P < 0.001. **E**–**G** Immunoblot analysis of K48 polyUb-modified proteins in siRNAs-transfected K562 (**E**), Jurkat (**F**), and H929 (**G**) cells with BTZ treatment. NS, NP-40 soluble; SS, NP-40 insoluble but SDS soluble. **H**–**J** FACS-based analysis of cell death in K562, Jurkat, and H929 cells that transiently transfected with CD317-specific siRNAs plus CNX-specific or control siRNAs in the presence or absence of BTZ. *P < 0.05; **P < 0.01; ***P < 0.001.

To check whether CNX is required for the aforementioned CD317 function, we knocked down CNX expression in CD317-knockdown tumor cells and found that calcium homeostasis (Fig. 6A–D) as well as proteostasis (Fig. 6E–G) were dramatically restored. As a result, the increased susceptibility of tumor cells to proteasome inhibitors caused by CD317 knockdown is no longer detectable after CNX reduction (Fig. 6H–J and Supplementary Fig. S6A–C). These results suggest that CNX is indispensable for CD317-mediated cell homeostasis and survival.

## Discussion

**Fig. 7:**
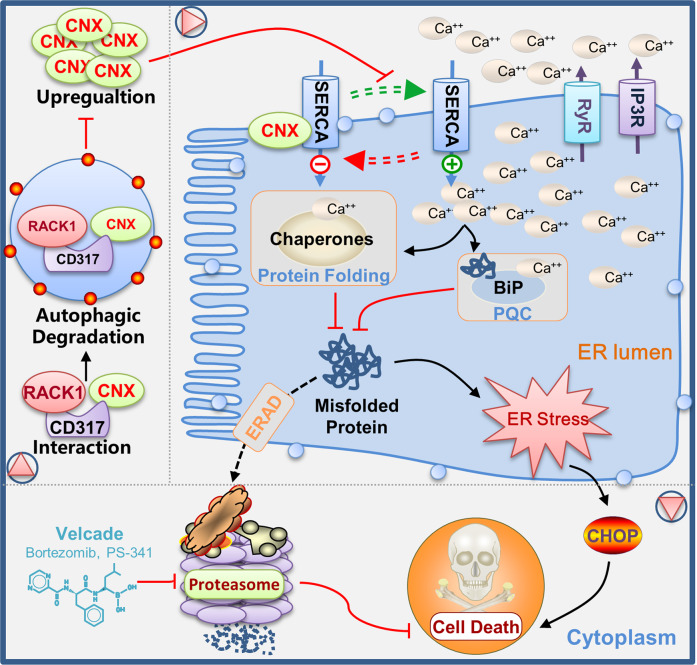
Proposed role of CD317 in proteostasis maintenance. CD317 expression is significantly increased in HM, leading to CNX degradation via RACK1-mediated autophagy. Thus, CNX expression is coordinated to fine-tune SERCA activity, which in turn maintains ER calcium homeostasis, creating a favorable environment for protein folding and quality control. As a result, CD317 knockdown impairs ER calcium homeostasis, making tumor cells especially sensitive to PIs-induced proteostasis collapse and eventual cell death.

Proteostasis is essential for tumor cell survival, yet it is frequently challenged by extrinsic unfavorable conditions and intrinsic stresses. As a result, proteostasis was considered to be the Achilles’ heel of tumor cells, and it has been extensively studied as a cancer therapy target. Here we reveal that CD317 is a key regulator of proteostasis in tumor cells and knocking it down makes tumor cells more susceptible to PIs-induced cell death (Fig. 7). This discovery has important implications for the development of more effective treatments for proteostasis-addicted tumors.

It was revealed that tumor cells require higher rates of protein synthesis for fast growth [32]. To avoid the buildup of misfolded proteins, the amounts of bulk protein synthesis must be tuned to the cell’s protein-folding capacity [33]. The endoplasmic reticulum (ER) functions as a protein-folding factory, with extensive quality and quantity control systems that monitor efficient and precise protein production. It is thus required for appropriate physiological homeostasis, such as calcium balance [34]. The amount of calcium in the ER is tightly regulated since it not only favors protein folding [23], but also fine-tunes ER quality control by stabilizing Bip-substrate complexes [35]. In this study, we found that CD317 knockdown decreases ER calcium while increasing misfolded protein accumulation, implying that CD317 is a novel regulator of ER calcium homeostasis that favors protein folding. Notably, cytosolic calcium levels are greater in CD317-knockdown cancer cells, hinting that CD317 knockdown causes Ca^2+^ sequestration in the cytoplasm. SERCAs (sarcoendoplasmic reticulum calcium transport ATPase) is the main pump that transports Ca^2+^ from the cytoplasm into ER [36], while CNX tightly regulating its activity. In this study, we discovered that CD317 interacts with CNX and subjects it to RACK1-mediated autophagic degradation, presumably boosting ER Ca^2+^ uptakes by favoring de-repression of SERCA activity (Fig. 7).

Autophagy is a cytoplasmic degradation system that is essential for stress adaptation and cellular quality control [37]. Autophagy is activated in response to and partially compensates for, proteasome impairment [38]. In multiple myeloma cells, a plastic SQSTM1/p62-dependent autophagic reserve has been implicated in maintaining proteostasis and determining PIs susceptibility [39]. Previous studies have shown that CD317 encourages both selective and non-selective autophagy [27, 40], while whether CD317 affects the response of cancer cells to PIs by modulating autophagic flux is yet to be determined. Our findings support prior evidence that autophagy has a protective function in tumor cell survival in response to PIs, and further demonstrate the role of CD317 in controlling this autophagic protective effect. CD317 knockdown impairs autophagy even in the presence of PIs (Fig. S5A–C), making tumor cells more vulnerable to BTZ-induced proteostasis collapse and cell death. More crucially, our study also provides a more comprehensive understanding of autophagy’s role in maintaining proteostasis: autophagy not only disposes of misfolded proteins, but it also constitutively optimizes the protein-folding compartment in tumor cells, a heretofore unrecognized function. In particular, CD317-mediated autophagy reduces CNX expression and hence elevates ER Ca^2+^ levels, favoring circumstances for protein folding. CD317 knockdown inhibits autophagy-mediated CNX degradation and ER Ca^2+^ uptake, promoting proteostasis collapse and making tumor cells more sensitive to PIs, while CNX reduction abolishes such effects, implying a vital role for CNX in such an integrated model underlying CD317-mediated proteostasis maintenance (Fig. 7). These findings help us better understand how autophagy regulates ER function and proteostasis.

Taken together, our findings identify CD317 as a key proteostasis regulator through coordinating the cooperation of the proteasome and autophagy in tumor cells, providing a potential target for overcoming PIs resistance or developing more effective proteostasis-targeting drugs.

## Supplementary information

Supplementary Methods and Figures

Checklist

## Data Availability

All relevant datasets supporting the conclusions of this study are available within the article and its additional files, or upon reasonable request from the corresponding authors.
