# Supplementary material for: CD317 maintains proteostasis and cell survival in response to proteasome inhibitors by targeting calnexin for RACK1-mediated autophagic degradation
Source: Cell Death Dis. 2023 May 20;14(5):333. doi: 10.1038/s41419-023-05858-1 (PMC10199885; doi:10.1038/s41419-023-05858-1)
Supplement: Supplementary file 1 — Supplementary Methods and Figures [file 41419_2023_5858_MOESM1_ESM.pdf]

Supplementary Methods and Figures

CD317 maintains proteostasis and cell survival in response to proteasome inhibitors by targeting calnexin for RACK1-mediated autophagic degradation

Jian Cheng[1,2], Guizhong Zhang[2,3,#], Tian Deng[2], Zhao Liu[2], Mengqi Zhang[2], Pengchao Zhang[2,4], Funmilayo O. Adeshakin[2,4], Xiangyun Niu[2,4], Dehong, Yan[2,3], Xiaochun Wan[2, 3, #], Guang Yu[1, #]

Supplementary Methods

Cell culture

HepG2, H1975, and MCF7 were preserved in-house and cultured in DMEM or RPMI-1640 medium (HyClone) supplemented with 10% FBS (HyClone) and 2 mmol/L L-glutamine (Gibco).

Cell viability

Twenty-four hours following transfection, K562 or Jurkat cells were seeded in triplicates in 96-well plates at 5,000 cells/well and maintained in a medium containing 10% FBS. Cells were stained with MTS (PR-G3580, Promega) at the indicated time points, and relative cell viability was determined by measuring the OD at an absorbance wavelength of 490 nm and 630 nm (reference wavelength).

Interaction modeling

Molecular docking was done with HADDOCK2.4 (https://wenmr.science.uu.nl/haddock2.4/) utilizing PDB data from AlphaFold Protein Structure Database (https://alphafold.ebi.ac.uk/) of CD317 (Q10589), RACK1 (P63244), and CNX (P27824). The docking data were then subjected to PDBePISA (https://www.ebi.ac.uk/msd-srv/prot_int/pistart.html) for the exploration of macromolecular interfaces. The modeling and image generation were performed with PyMOL2.5 software (https://pymol.org/2/).

FACS-based autophagy detection

48 hours following transfection, cells were collected and labeled with CYTO-ID autophagy detection reagent (ENZ-51031-0050) according to manufacturers'

instructions (Enzo Life Sciences, Inc.), then analyzed without washing by flow cytometry (CytoFLEX, BECKMAN).

Supplementary Figures

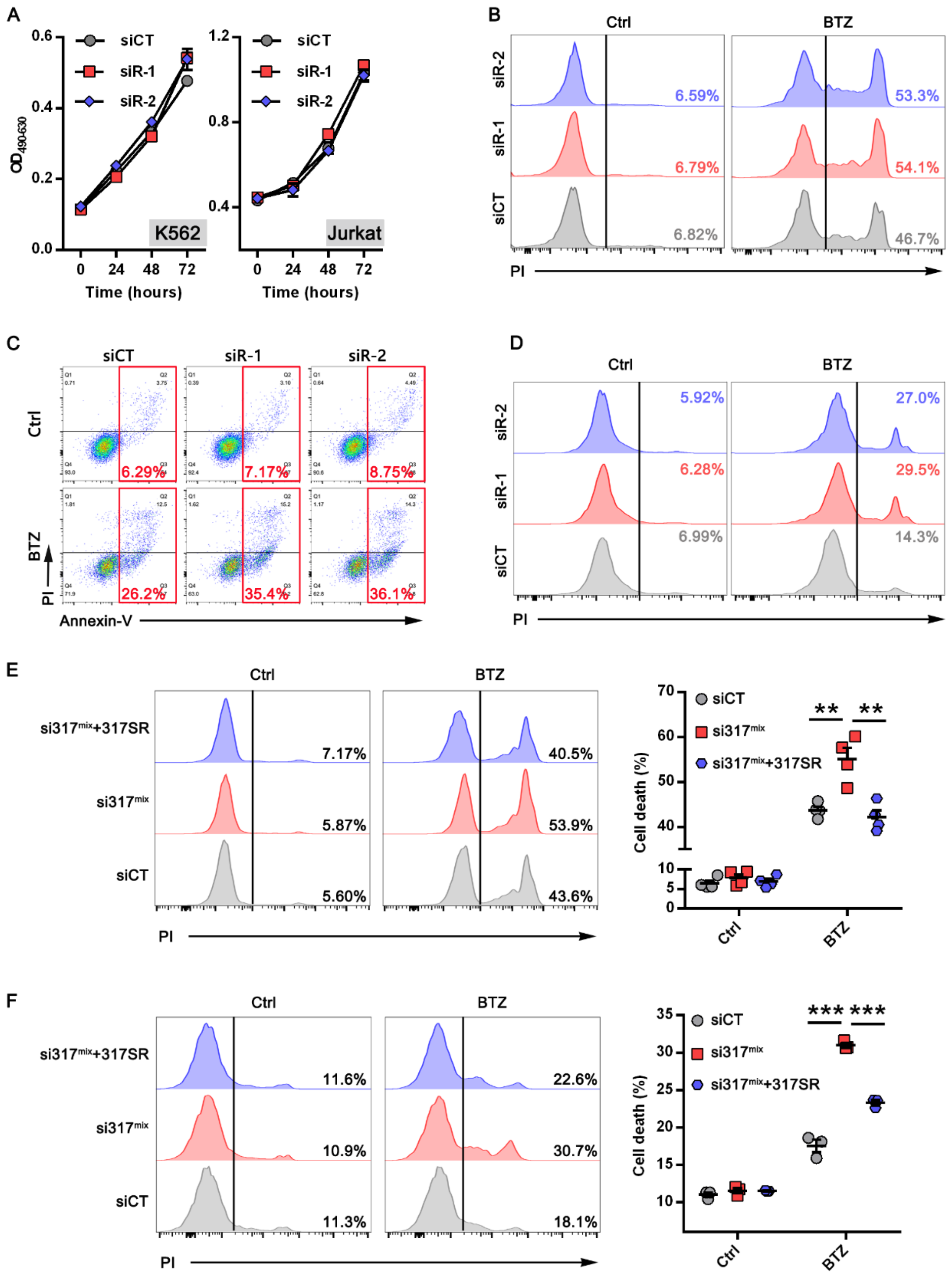

Figure S1. Role of CD317 in the proliferation and cell death of HM cells.

(**A**), Proliferation of K562 and Jurkat cells transiently transfected with control (siCT) or CD317 siRNA (siR-1 or siR-2). (**B-D**), Representative FACS images showing cell

death of K562 (B), Jurkat (C), and H929 (D) cells transiently transfected with CD317-specific siRNAs (siR-1 or siR-2) or control siRNAs. (**E**), Representative FACS graphs (left) and quantification (right) of cell death in Jurkat cells transfected with indicated siRNAs plus siRNA-resistant CD317 plasmids in the presence or absence of BTZ treatment; (**F**), Representative FACS graphs (left) and quantification (right) of cell death in K562 cells transfected with indicated siRNAs plus siRNA-resistant CD317 plasmids in the presence or absence of BTZ treatment; For E-F, $**P < 0.01$; $***P < 0.001$.

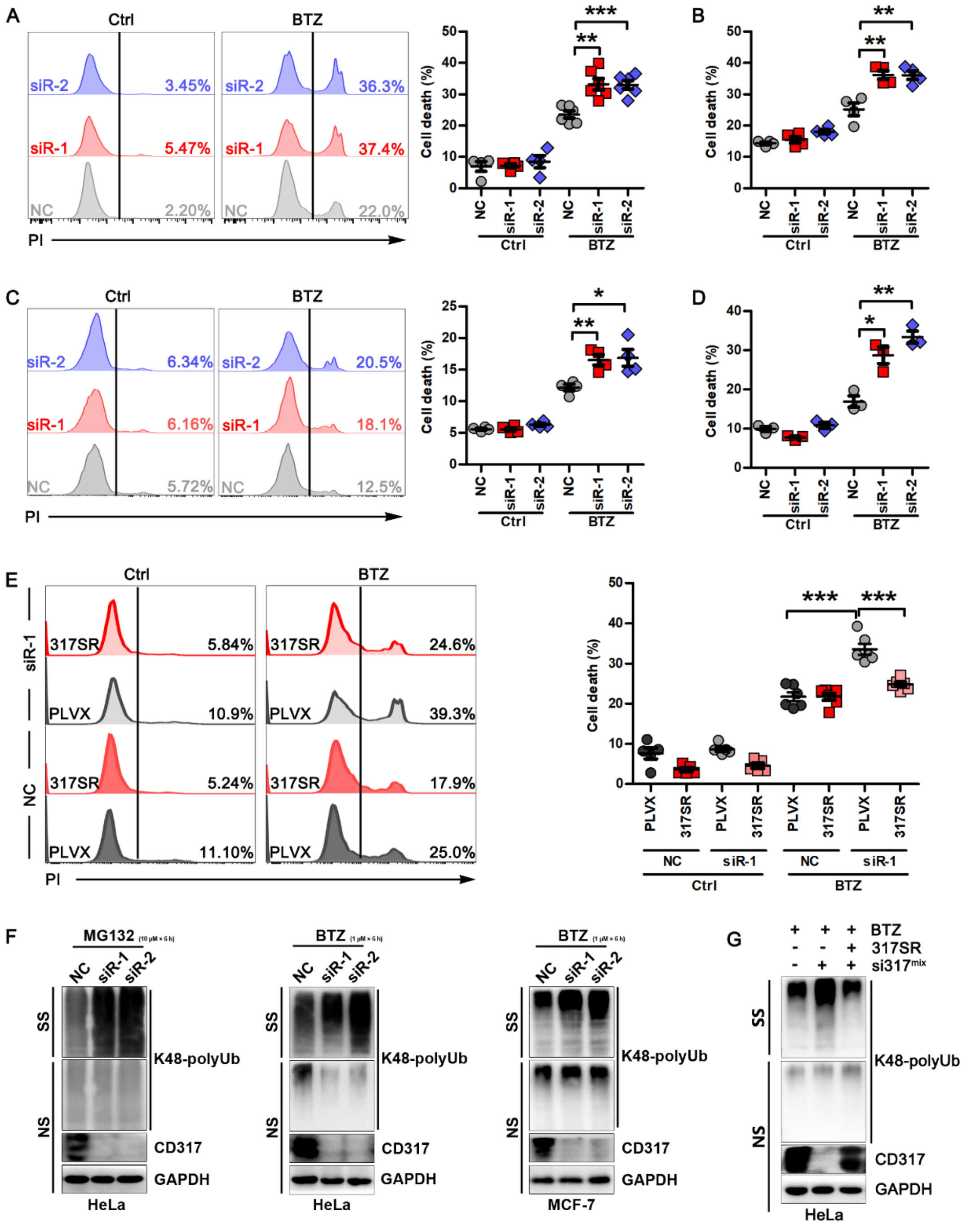

Figure S2. CD317 knockdown promotes BTZ-induced cell death and proteostasis collapse in solid tumor cells.

(**A**), Representative FACS graphs (left) and quantification (right) of cell death in HeLa cells transfected with indicated siRNAs in the presence or absence of BTZ treatment; (**B**), FACS-based cell death analysis of siRNA-transfected MCF-7 in the presence or

absence of BTZ treatment; (**C**), Representative FACS graphs (left) and quantification (right) of cell death in HepG2 cells transfected with indicated siRNAs in the presence or absence of BTZ treatment; (**D**), FACS-based cell death analysis of siRNA-transfected H1975 in the presence or absence of BTZ treatment; (**E**), Representative FACS graphs (left) and quantification (right) of cell death in HeLa cells transfected with indicated siRNAs plus siRNA-resistant CD317 plasmids in the presence or absence of BTZ treatment; For A-E, $^{*}P < 0.05$; $^{**}P < 0.01$; $^{***}P < 0.001$; (**F**), Immunoblot analysis of K48 polyUb-modified proteins in CD317 siRNA-transfected HeLa and MCF-7 cells treated with BTZ or MG132 treatment. NS, NP-40 soluble; SS, NP-40 insoluble but SDS soluble; (**G**), Immunoblot analysis of K48 polyUb-modified proteins in CD317-knockdown Hela cells after forced expression of siRNA-resistant CD317.

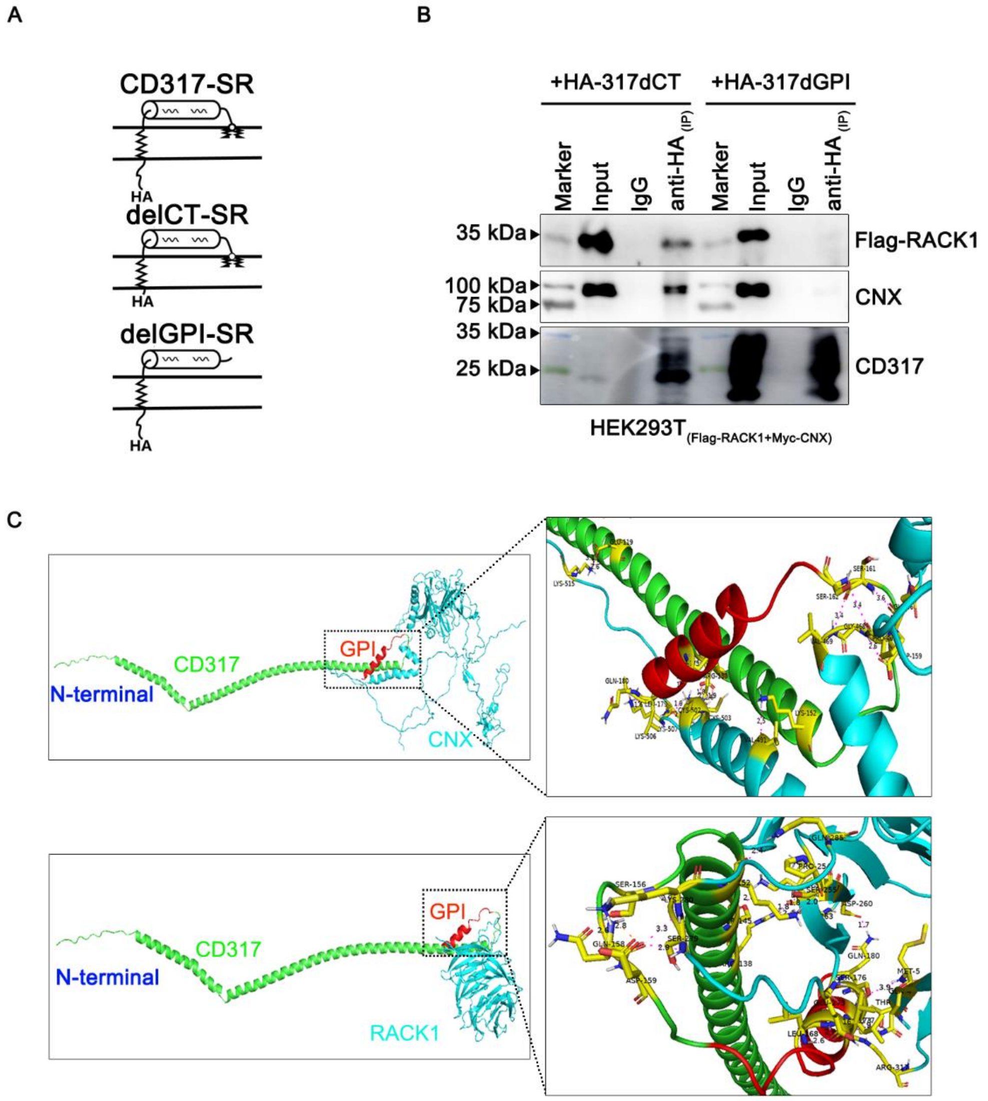

Figure S3. Mapping and modeling of the interaction between CD317 and CNX, RACK1.

(**A**), Schematic representation of the CD317 mutants panel. (**B**), Immunoblot analysis of the interaction between CD317 mutants with CNX and RACK1. HEK293T cells were transfected with plasmids encoding Flag-RACK1 and Myc-CNX together with HA-CD317 mutants, followed by IP with anti-HA beads and immunoblot analysis with the indicated antibodies. (**C**), Molecular modeling showing potential interaction between CD317 and CNX or RACK1.

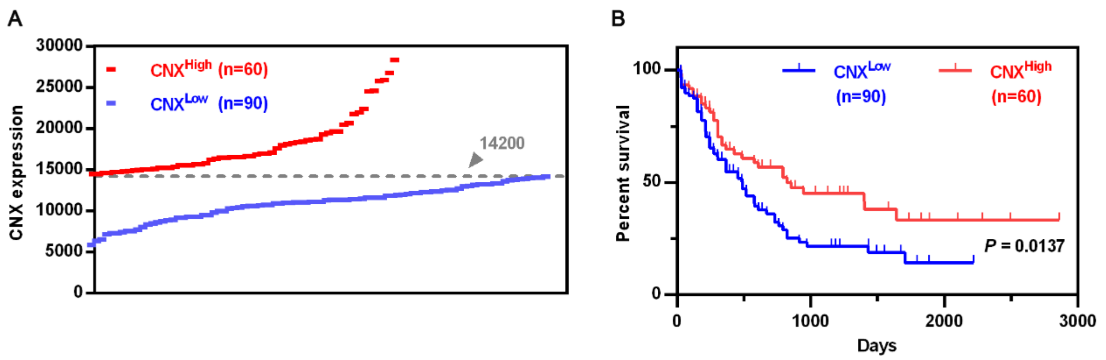

Figure S4. Kaplan–Meier survival analysis for CNX expression in LAML patients from Oncolnc database.

The overall survival were compared between CNX high (n = 60) and low expression (n = 90) in LAML patients from Oncolnc database.

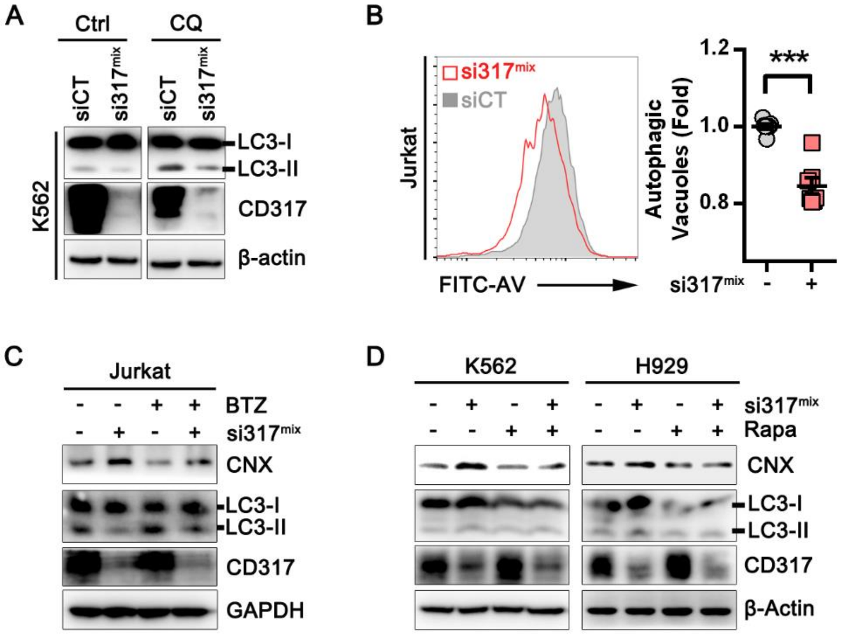

Figure S5. CD317 knockdown reduces autophagy flux and thus inhibits CNX degradation

(**A**), Immunoblot analysis of LC3 proteins in CD317-knockdown (si317mix) and control (siCT) K562 cells treated with or without Chloroquine (CQ, 50 µM, 3 h). (**B**), Representative FACS graphs (left) and quantification (right) of autophagic vacuoles in

CD317-knockdown Jurkat (si317mix) and the corresponding control cells (siCT); ****P* ＜0.001; (**C**), Immunoblot analysis of CNX and LC3 proteins in CD317-knockdown Jurkat cells treated with or without BTZ (1 μM, 6 h). (**D**), Immunoblot analysis of CNX proteins in cells treated with or without Rapamycin (Rapa) as indicated.

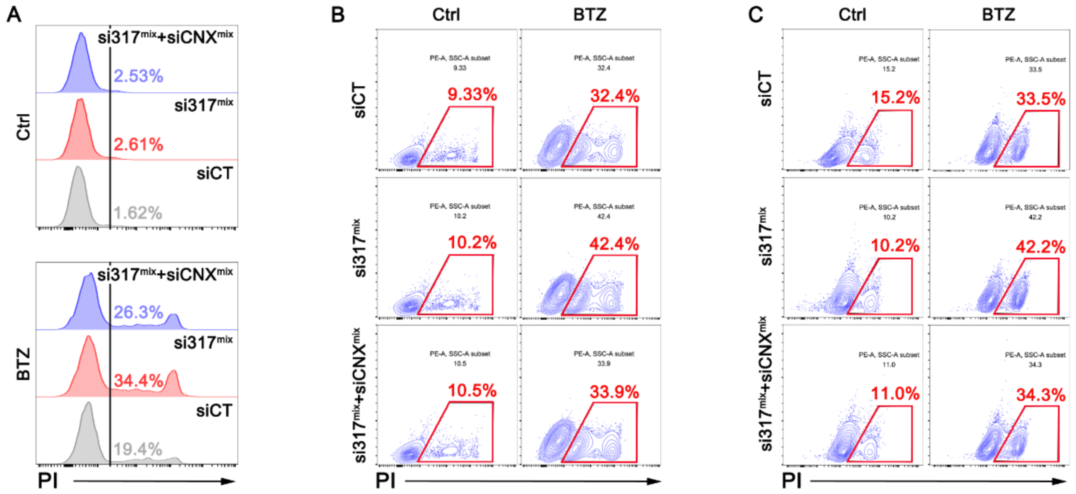

Figure S6. CNX is indispensable for CD317-mediated cell survival

(**A-C**), Representative FACS graphs of cell death in K562 (A), Jurkat (B), and H929 (C) cells that transiently transfected with CD317-specific siRNAs plus CNX-specific or control siRNAs in the presence or absence of BTZ.

A

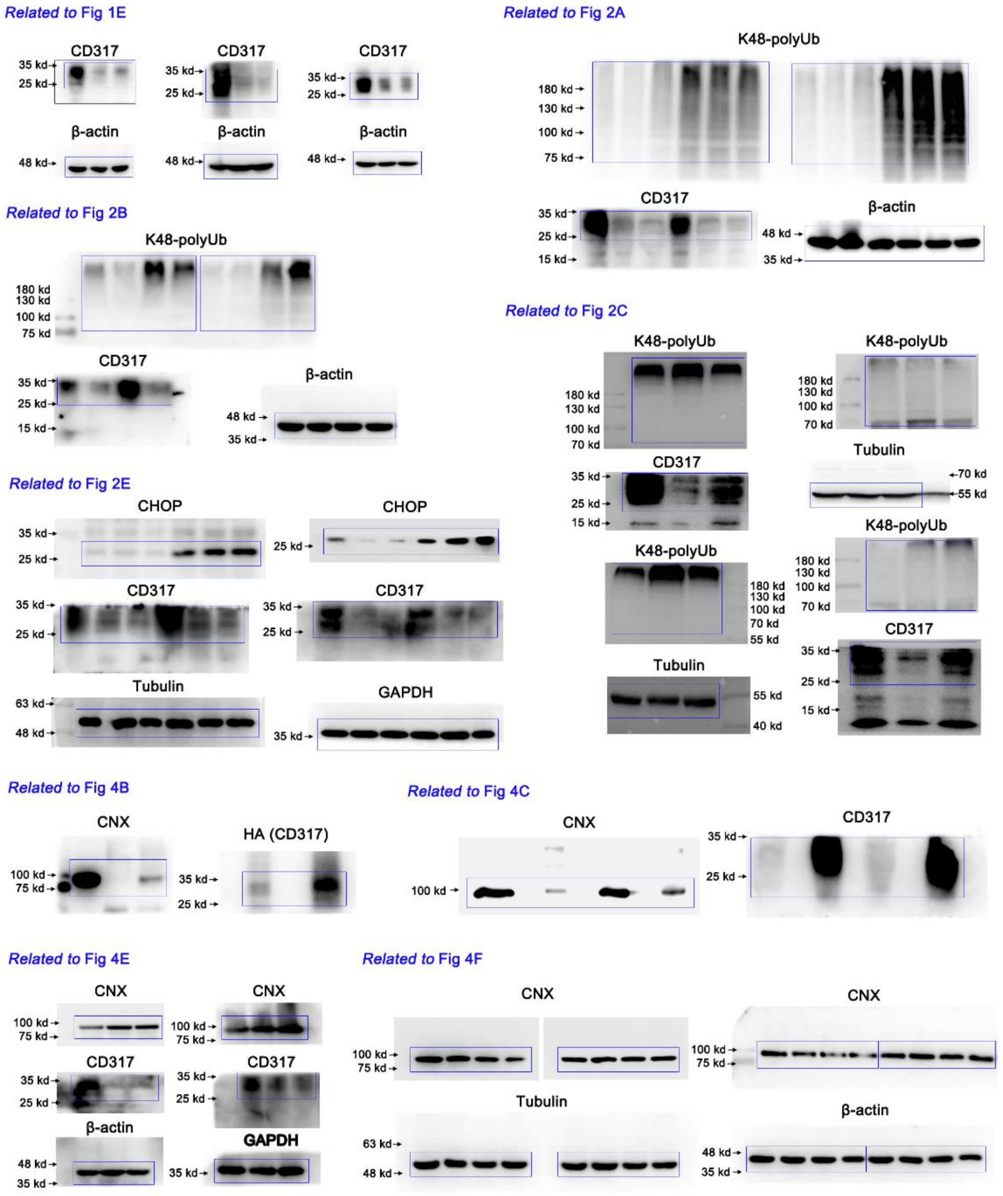

Related to Fig 1E
CD317
35 kd
25 kd
β-actin
48 kd
Related to Fig 2A
K48-polyUb
180 kd
130 kd
100 kd
75 kd
CD317
35 kd
25 kd
15 kd
β-actin
48 kd
35 kd
Related to Fig 2B
K48-polyUb
180 kd
130 kd
100 kd
75 kd
CD317
35 kd
25 kd
15 kd
β-actin
48 kd
35 kd
Related to Fig 2C
K48-polyUb
180 kd
130 kd
100 kd
70 kd
CD317
35 kd
25 kd
15 kd
Tubulin
70 kd
55 kd
40 kd
Related to Fig 2E
CHOP
35 kd
25 kd
CD317
Tubulin
63 kd
48 kd
GAPDH
Related to Fig 4B
CNX
100 kd
75 kd
HA (CD317)
35 kd
25 kd
Related to Fig 4C
CNX
100 kd
CD317
35 kd
25 kd
Related to Fig 4E
CNX
100 kd
75 kd
CD317
35 kd
25 kd
β-actin
48 kd
35 kd
GAPDH
Related to Fig 4F
CNX
Tubulin
63 kd
48 kd
β-actin
48 kd
35 kd

B

Related to Fig 5B

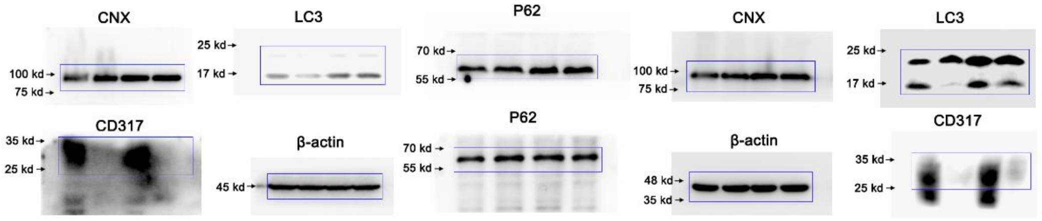

Related to Fig 5D

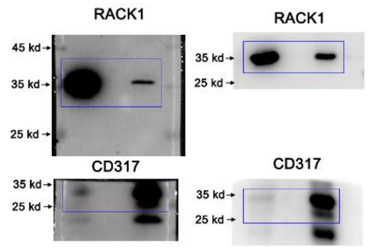

Related to Fig 5E

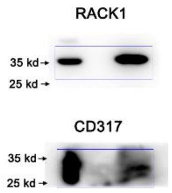

Related to Fig 5F

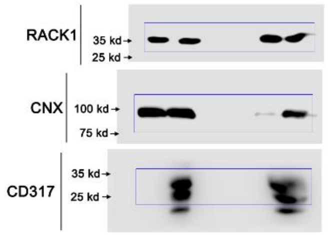

Related to Fig 5G

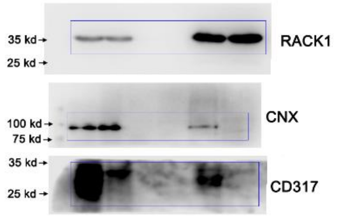

Related to Fig 5H

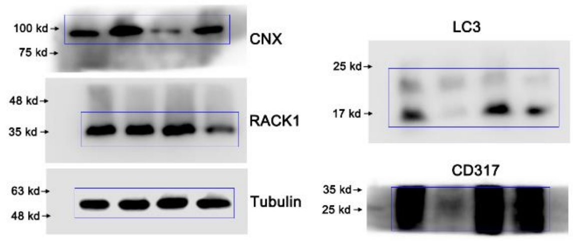

Related to Fig 6E

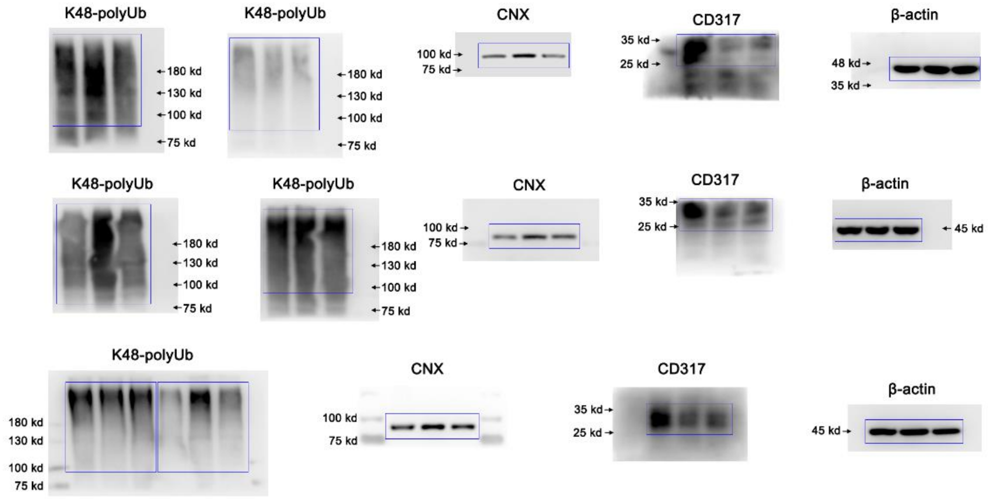

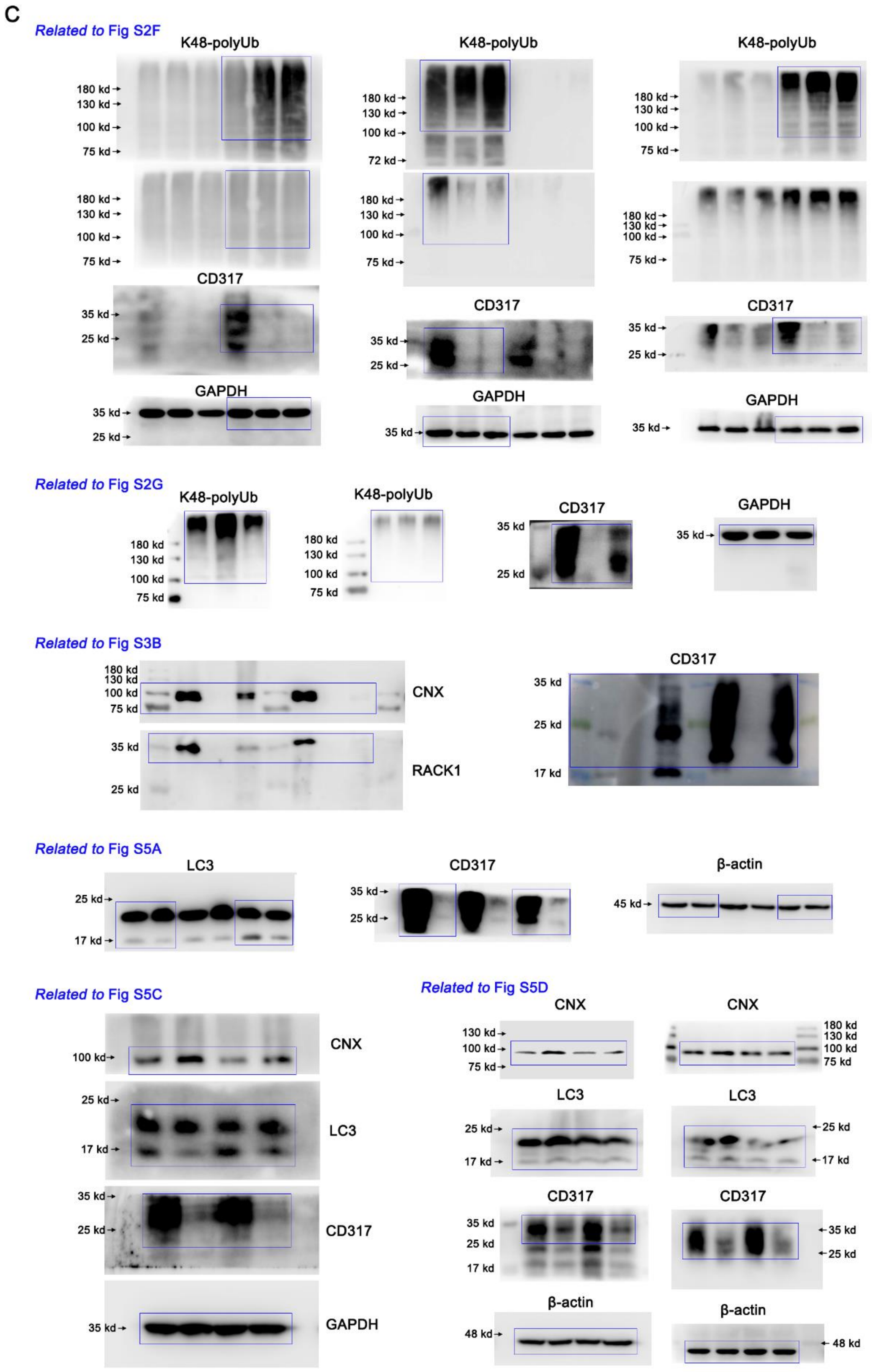

Figure S7. Full Western blots

(**A**), Uncropped immunoblots related to Figure 1-4; (**B**), Uncropped immunoblots related to Figure 5-6; (**C**), Uncropped immunoblots related to Supplemental Figures.
